# Pharmacotherapeutic profile, polypharmacy and its associated factors in a cohort of people living with HIV in Brazil

**DOI:** 10.1186/s12981-023-00548-6

**Published:** 2023-08-21

**Authors:** Robson Pierre Nascimento da Silva, Luana M. S. Marins, Lusiele Guaraldo, Paula Mendes Luz, Sandra W. Cardoso, Ronaldo I. Moreira, Vanessa da Gama Oliveira, Valdilea G. Veloso, Beatriz Grinsztejn, Rita Estrela, Thiago S. Torres

**Affiliations:** 1 Laboratório de Pesquisa Clínica em DST/AIDS, Instituto Nacional de Infectologia Evandro Chagas, Fundação Oswaldo Cruz (INI-Fiocruz), Rio de Janeiro, Brazil; 2 Laboratório de Pesquisa Clínica em Doenças Febris Agudas, Instituto Nacional de Infectologia Evandro Chagas, Fundação Oswaldo Cruz (INI-Fiocruz), Rio de Janeiro, Brazil; 3 Serviço de Farmácia (Sefarm), Instituto Nacional de Infectologia Evandro Chagas, Fundação Oswaldo Cruz (INI-Fiocruz), Rio de Janeiro, Brazil; 4 Laboratório de Farmacometria, Faculdade de Farmácia, Universidade Federal do Rio de Janeiro (UFRJ), Rio de Janeiro, Brazil; 5 Av Brasil 4365 Manguinhos, 21045-360 Rio de Janeiro, Brazil

**Keywords:** HIV, Antiretroviral therapy, Polypharmacy, Pharmacy, Brazil

## Abstract

**Background:**

The increased survival provided by the access, development, and evolution of antiretroviral drugs (ARV) greatly increased the life expectancy of people living with HIV (PWH). This has also led to an increased occurrence of diseases or morbidities related to aging. In individuals with multiple comorbidities, the simultaneous use of multiple medications, also known as polypharmacy, is common, and rational use of medications is essential. This study aims to describe the pharmacotherapeutic profile, estimate the prevalence of polypharmacy and identify factors associated with polypharmacy in a cohort of adult PWH from a referral unit in Rio de Janeiro, Brazil.

**Methods:**

Cross-sectional study including PWH on ARV who received at least one medical prescription (outpatient/hospitalized) in 2019. We described the proportion of prescribed medications according to ARV and Anatomical Therapeutic Chemical (ATC) classes stratified by age (< 50 vs. ≥50 years). Polypharmacy was defined as ≥ 5 medications prescribed beyond ARV. Logistic regression models assessed demographic and clinical factors associated with polypharmacy.

**Results:**

A total of 143,306 prescriptions of 4547 PWH were analyzed. Median age was 44.4 years (IQR:35.4–54.1) and 1615 (35.6%) were ≥ 50 years. A total of 2958 (65.1%) participants self-identified as cisgender man, 1365 (30.0%) as cisgender woman, and 224 (4.9%) as transgender women. Most self-declared Black/*Pardo* (2582; 65.1%) and 1984 (44.0%) completed elementary education or less. Median time since HIV diagnosis was 10.9 years (IQR:6.2–17.7). Most frequently prescribed concomitant medications were nervous system (64.8%), antiinfectives for systemic use (60.0%), alimentary tract and metabolism (45.9%), cardiovascular system (40.0%) and respiratory system (37.1%). Prevalence of polypharmacy was 50.6% (95%CI: 49.2–52.1). Model results indicated that being older, self-identify as cisgender woman, having less education and longer time since HIV diagnosis increased the odds of polypharmacy.

**Conclusions:**

We found high rates of polypharmacy and concomitant medication use in a cohort of PWH in Brazil. Targeted interventions should be prioritized to prevent interactions and improve treatment, especially among individuals using central nervous system and cardiovascular medications, as well as certain groups such as cisgender women, older individuals and those with lower education. Standardized protocols for continuous review of patients’ therapeutic regimens should be implemented.

**Supplementary Information:**

The online version contains supplementary material available at 10.1186/s12981-023-00548-6.

## Background

According to Joint United Nations Programme on HIV/AIDS (UNAIDS), around 38.4 million people were living with HIV and 28.2 million people living with HIV (PWH) had access to antiretroviral drugs (ARV) in 2021 [1]. In Brazil, the AIDS detection rate has declined, from 22.0/100,000 inhabitants in 2012 to 14.1/100,000 in 2020, representing a decrease of 35.7% [2]. This is likely a result of public policies that allowed free-of-charge access to ARV through the Unified Health System (*Sistema Único de Saúde*, SUS) since 1996 [3], and provision of combination HIV prevention including condoms, post-exposure prophylaxis (PEP), pre-exposure prophylaxis (PrEP), test and treat, and ARV for all people newly diagnosed with HIV since 2014 [4]. In addition, the introduction of integrase inhibitors (INSTI) has greatly improved the effectiveness of ARV treatment. Evidence from a systematic review strongly suggested that dolutegravir based regimen are superior to efavirenz based regimen in a variety of outcomes, such as efficacy, safety and tolerability [5].

The increased survival provided by the access, development and evolution of ARV greatly increased the life expectancy of PWH. In Latin America, overall life expectancy increased from 31.0 (95% CI: 29.3–32.8) to 69.5 years (95% CI: 67.2–71.8) from 2003–2008 to 2013–2017, similar to life expectancy of the general population [6]. However, PWH have an increased burden of age-associated comorbidities compared with individuals not living with HIV [7, 8]. This may be explained by an accelerated or accentuated aging process [9], resulting from several factors such as HIV infection itself, ARV treatment, bacterial translocation, chronic viral co-infections and lifestyle/behavioral factors [8, 10]. There is evidence that ARV can disrupt several cellular processes, including autophagy, which has been linked to the premature aging of people on ARV therapy [11].

In individuals with multiple comorbidities, the simultaneous use of multiple medications, also known as polypharmacy, is common, and rational use of medications is essential [12]. Inadequate polypharmacy may reduce the expected clinical benefit of medications, and increase the risk of drug interactions, toxicity, adherence problems, and hospitalization [12]. Caution should be taken regarding polypharmacy among PWH, as important interactions between ARV and concomitant medications are common [13, 14]. Moreover, polypharmacy among PWH is associated with worse self-perception of health [14] and worse health outcomes regardless of existing comorbidities [15].

It is important to understand the profile of medications used by PWH to increase effectiveness, safety and therapeutic success [16]. This study aims to describe the pharmacotherapeutic profile, estimate the prevalence of polypharmacy and identify factors associated with polypharmacy in a cohort of adult PWH from a referral unit in Rio de Janeiro, Brazil.

## Methods

### Study design

Observational, retrospective, cross-sectional study including PWH aged 18 years or older followed at the Instituto Nacional de Infectologia Evandro Chagas, Fundação Oswaldo Cruz (INI/Fiocruz) who were currently using ARV. Patients were included if there was at least one medical prescription (outpatient or hospitalized) for them in INI/Fiocruz electronic system during the year 2019. The source of information for the study was based exclusively on medical records and medical prescriptions.

#### Main outcomes

This study has three main outcomes: (1) number of PWH using ARV; (2) number of PWH using concomitant medications; (3) polypharmacy. ARV were classified according to pharmacological class: nucleoside reverse transcriptase inhibitors (NRTI): abacavir, emtricitabine, lamivudine, tenofovir, zidovudine; non-nucleoside reverse transcriptase inhibitors (NNRTI): efavirenz, etravirine; protease inhibitors (PI): atazanavir, darunavir, lopinavir, ritonavir, tipranavir; fusion inhibitor (IF): enfurvitide (T20); integrase strand transfer inhibitors (INSTI): dolutegravir, raltegravir; CCR5 co-receptor antagonists: maraviroc. Concomitant prescribed medications (except for ARV) were classified according to the second level of classification of the World Health Organization (WHO) Anatomical Therapeutic Chemical (ATC) index, version 2021 [17]. Polypharmacy was defined as the prescription of five or more concomitant medications (all prescribed drugs except ARV) [18].

### Additional variables and definitions

We evaluated sociodemographic variables (age, gender, race and education) and time since HIV diagnosis. Age (years) was calculated in reference to December 31, 2019 (date of study closure), described as median and interquartile range (IQR), and in age strata (18–39, 40–49, 50–59, 60–69, ≥ 70 years). Gender was stratified into cisgender man, cisgender woman and transgender woman. Race was stratified in Black, *Pardo* (Mix-black) and White, according to Brazilian standard classification [19]. Education was defined at four levels: less than elementary (≤ 4 years), elementary (5–8 years), secondary (9–11 years) and higher than secondary (≥ 12 years). Time since HIV diagnosis and time since ARV initiation were calculated in reference to December 31, 2019, presented as median and IQR, and dichotomized in ≤ 10 years and > 10 years.

### Data analysis

We described the proportion of PWH receiving prescribed medications overall and stratified according to age group (< 50 vs. ≥ 50 years). We assessed age differences using chi-square tests. We used unadjusted and adjusted logistic regression analyses to assess factors associated with polypharmacy. Variables with p < 0.05 threshold in unadjusted analyses (OR: odds ratio) were retained in final multivariate model (aOR: adjusted odds ratio). All analyses were performed on R version 4.0.4 [20].

## Results

A total of 143,306 prescriptions of 4547 PWH followed at INI-Fiocruz received at least one medical prescription and were included in this study. The median age was 44.4 years (IQR: 35.4–54.1) and 1615 (35.5%) were 50 years or older (Table 1). A total of 2958 (65.1%) participants self-identified as cisgender man, 1365 (30.0) as cisgender woman and 224 (4.9%) as transgender women. Over half self-declared as Black or *Pardo* (2582; 56.8%) and 1079 (23.9%) had less than elementary education. The median time since HIV diagnosis was 10.9 years (IQR: 6.2–17.7); 2472 (54.4%) and 1932 (42.7%) participants were diagnosed with HIV and initiated ARV more than 10 years ago, respectively. Only 123 (2.7%) participants were hospitalized during the study period, and 259 (5.7%) were on ARV therapy for one year or less.

**Table 1:** Characteristics of people living with HIV who received at least one medical prescription in a referral center in Rio de Janeiro, Brazil, 2019 (N = 4547)

	N = 4547 (%)
Age (years)	
Median (IQR)	44.4 (35.4, 54.1)
18–39	1726 (38.0)
40–49	1206 (26.5)
50–59	1061 (23.3)
60–69	459 (10.1)
≥ 70	95 (2.1)
Gender	
Cisgender man	2958 (65.1)
Cisgender woman	1365 (30.0)
Transgender woman	224 (4.9)
Race	
Black	922 (20.3)
*Pardo* (Mixed-race)	1660 (36.5)
White	1965 (43.2)
Education	
Less than elementary	1079 (23.9)
Elementary	905 (20.1)
Secondary	1858 (41.2)
Superior	671 (14.9)
Time since HIV diagnosis (years)	
Median (IQR)	10.9 (6.2, 17.7)
≤ 10	2075 (45.6)
> 10	2472 (54.4)
Time since ARV initiation (years)	
Median (IQR)	8.7 (5.0, 14.2)
≤ 10	2593 (57.3)
> 10	1932 (42.7)

IQR: interquartil range; ARV: antiretroviral drug

Most of participants received prescriptions with NRTI drugs (4348; 95.6%), followed by PI (2023; 44.5%), INSTI (1999; 44.0%) and NNRTI (1638; 36.0%) (Table 2). Lamivudine (4337; 95.4%), tenofovir (3718; 81.8%), dolutegravir (1979; 43.5%) and efavirenz (1551; 34.1%) were the most prescribed ARV. Tenofovir/lamivudine/efavirenz co-formulation was prescribed to 1338 (29.4%) participants. Compared to participants aged < 50 years, older participants (≥ 50 years) received more prescriptions with any PI drug (56.4% vs. 37.9%, p < 0.001), etravirine (2.3% vs. 0.8%, p < 0.001) and maraviroc (1.7% vs. 0.4%, p < 0.001).

**Table 2:** Proportion of participants who received antiretroviral prescriptions according to age strata (< 50 vs. ≥ 50 years) in a referral center in Rio de Janeiro, Brazil, 2019 (N = 4547)

	Total N = 4547 (%)	< 50 years N = 2932 (%)	≥ 50 years N = 1615 (%)	P value
**NRTI** ^**1**^	4348 (95.6)	2858 (97.5)	1490 (92.3)	< 0.001
Lamivudine	4337 (95.4)	2853 (97.3)	1484 (91.9)	< 0.001
Tenofovir	3718 (81.8)	2608 (88.9)	1110 (68.7)	< 0.001
Abacavir	343 (7.5)	151 (5.2)	192 (11.9)	< 0.001
Zidovudine	304 (6.7)	122 (4.2)	182 (11.3)	< 0.001
**NNRTI** ^**1**^	1638 (36.0)	1067 (36.4)	571 (35.4)	0.49
Efavirenz	1551 (34.1)	1031 (35.2)	520 (32.2)	0.044
Etravirine	59 (1.3)	22 (0.8)	37 (2.3)	< 0.001
Nevirapine	19 (0.4)	10 (0.3)	9 (0.6)	0.28
Rilpivirine	10 (0.2)	5 (0.2)	5 (0.3)	0.34
**INSTI**	1999 (44.0)	1302 (44.4)	697 (43.2)	0.42
Dolutegravir	1979 (43.5)	1291 (44.0)	688 (42.6)	0.35
Raltegravir	36 (0.8)	23 (0.8)	13 (0.8)	0.94
**PI**	2023 (44.5)	1112 (37.9)	911 (56.4)	< 0.001
Ritonavir	2023 (44.5)	1112 (37.9)	911 (56.4)	< 0.001
Darunavir	1180 (26.0)	616 (21.0)	564 (34.9)	< 0.001
Atazanavir	909 (20.0)	529 (18.0)	380 (23.5)	< 0.001
Lopinavir	12 (0.3)	6 (0.2)	6 (0.4)	0.29
Tipranavir	6 (0.1)	0 (0)	6 (0.4)	< 0.001
**FI**				
Enfurvitide	8 (0.2)	4 (0.1)	4 (0.1)	0.39
**CCR5 antagonist**				
Maraviroc	40 (0.9)	13 (0.4)	27 (1.7)	< 0.001

NRTI: nucleoside reverse transcriptase inhibitor; NNRTI: nucleoside/nucleotide reverse transcriptase inhibitors; INSTI: integrase inhibitors; PI: protease inhibitors; FI: fusion inhibitors. ^1^Tenofovir/Lamivudine/Efavirenz co-formulation was considered to calculate proportion of users

**Fig. 1:**
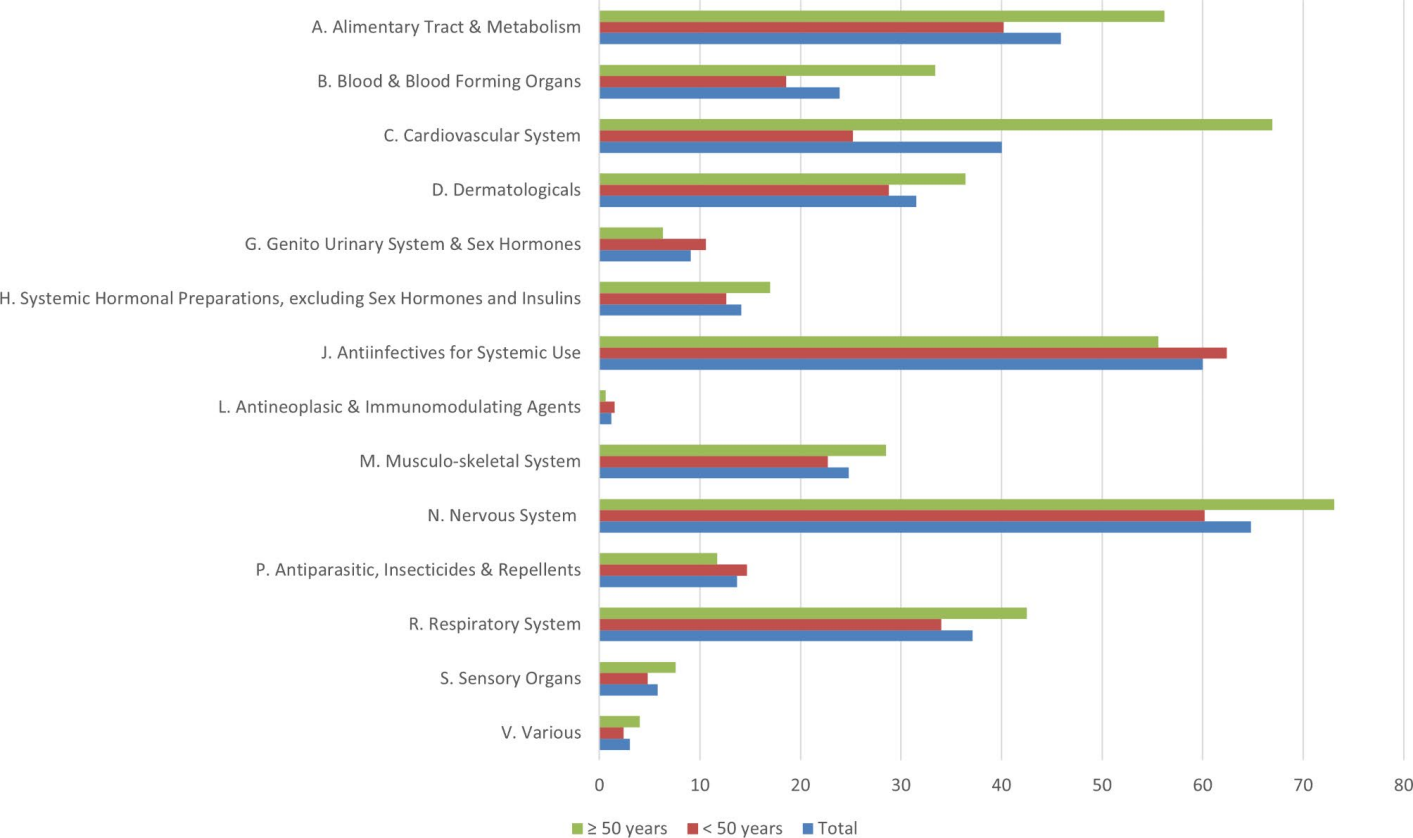
Proportion of people living with HIV (%) receiving concomitant medication prescriptions according to the Anatomical Therapeutical Classification (ATC) stratified by age (< 50 vs. ≥50 years) in a referral center in Rio de Janeiro, Brazil, 2019

Concomitant medications groups most frequently prescribed were nervous system (Class N: e.g. analgesics and antiepileptics) (64.8%), antiinfectives for systemic use (Class J: e.g. antibacterials and antimicotics) (60.0%), alimentary tract and metabolism (Class A: e.g. acid related disorders and functional gastrointestinal disorders agents) (45.9%), cardiovascular system (Class C: e.g. lipid modifying agents, diuretics and beta blocking agents) (40.0%) and respiratory system (Class R: e.g. antihistamines for systemic use) (37.1%) (Supplementary Table 1). Concomitant medications were more frequently prescribed to older participants (≥ 50 years) compared to participants with less than 50 years in nine out of 14 ATC classes (Fig. 1).

**Fig. 2:**
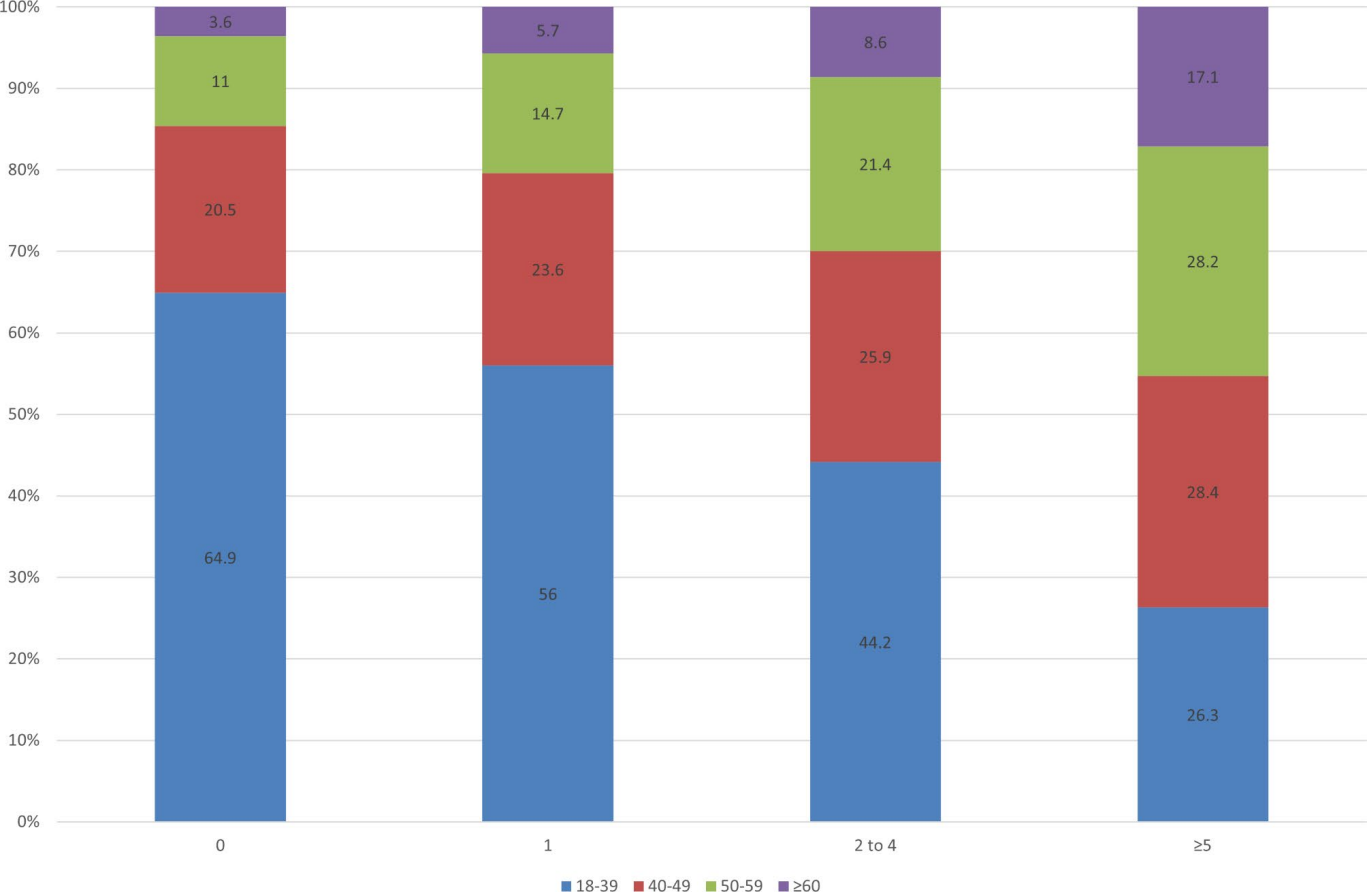
Proportion of participants according to the number of concomitant medications prescriptions stratified by age (years) in a referral center in Rio de Janeiro, Brazil, 2019 (N = 4547)

Prevalence of polypharmacy (≥ 5 concomitant medications) was 50.6% (95%CI: 49.2–52.1). Only 336 (7.4%) participants received no concomitant medication, while 495 (10.9%) and 1414 (31.1%) received one and 2–4 concomitant medications, respectively. Number of concomitant medications prescribed increased with age (p < 0.001) (Fig. 2). Prevalence of polypharmacy among PWH according to age was 35.1% (18–39 years), 54.2% (40–49 years), 61.2% (50–59 years) and 70.9% (≥ 60 years).

On adjusted logistic regression model, odds of polypharmacy increased with age: aOR 1.79 (95%CI: 1.33–2.44) for 40–49 years, aOR 2.80 (95%CI: 1.90–4.21) for 50–59 years, and aOR 4.41 (95%CI: 2.45–8.67) for ≥ 60 years (Table 3). Additionally, self-identifying as cisgender woman (aOR 1.75, 95%CI: 1.27–2.47) compared to cisgender man, completing less than elementary (aOR 4.09, 95%CI: 2.77-6,14), elementary (aOR 3.44, 95%CI: 2.36–5.09) or secondary education (aOR 2.60, 95%CI: 1.96–3.43) compared to higher than secondary education, and having more than 10 years since HIV diagnosis compared to less than 10 years (aOR 1.78, 95%CI: 1.35–2.35) increased the odds of polypharmacy.

**Table 3:** Factors associated with polypharmacy in a referral center in Rio de Janeiro, Brazil, 2019 (N = 4547)

	Polypharmacy (≥ 5 concomitant drugs)		Bivariate models		Multivariate model	
Total	**No 2245 (49.4%)**	**Yes 2302 (50.6%)**	OR (95%CI)	P-value	aOR (95%CI)	P-value
Age (years)						
18–39	1120 (64.9)	606 (35.1)	Ref.		Ref.	
40–49	552 (45.8)	654 (54.2)	2.38 (1.81–3.18)	< 0.001	1.79 (1.33–2.44)	< 0.001
50–59	412 (38.8)	649 (61.2)	4.00 (2.83–5.80)	< 0.001	2.80 (1.90–4.21)	< 0.001
≥ 60	161 (29.1)	393 (70.9)	6.53 (3.79–12.43)	< 0.001	4.41 (2.45–8.67)	< 0.001
Gender						
Cisgender man	1675 (56.6)	1283 (43.4)	Ref.		Ref.	
Cisgender woman	452 (33.1)	913 (66.9)	2.73 (2.02–3.78)	< 0.001	1.75 (1.27–2.47)	< 0.001
Transgender woman	118 (52.7)	106 (47.3)	1.02 (0.65–1.68)	0.95	1.07 (0.66–1.82)	0.80
Race						
Black	455 (49.3)	467 (50.7)	0.92 (0.69–1.24)	0.58	NA	NA
*Pardo* (Mixed-race)	829 (49.9)	831 (50.1)	0.98 (0.76–1.26)	0.89	NA	NA
White	961 (48.9)	1004 (51.1)	Ref.		NA	NA
Education						
Less than elementary	376 (34.8)	703 (65.2)	5.06 (3.49–7.48)	< 0.001	4.09 (2.77–6.14)	< 0.001
Elementary	387 (42.8)	518 (57.2)	3.71 (2.59–5.40)	< 0.001	3.44 (2.36–5.09)	< 0.001
Secondary	1013 (54.5)	845 (45.5)	2.24 (1.71–2.93)	< 0.001	2.60 (1.96–3.43)	< 0.001
Higher than secondary	458 (68.3)	213 (31.7)	Ref.		Ref.	
Time since HIV diagnosis (years)						
≤ 10	1255 (60.5)	820 (39.5)	Ref.		Ref.	
> 10	990 (40.0)	1482 (60.0)	3.00 (2.36–3.83)	< 0.001	1.84 (1.23–2.84)	< 0.001
Time since ARV initiation (years)						
≤ 10	1484 (66.5)	1109 (48.3)	Ref.		Ref.	
> 10	747 (33.5)	1185 (51.7)	2.88 (2.21–3.79)	< 0.001	0.97 (0.60–1.53)	0.91

## Discussion

In this study, we described the pharmacotherapeutic profile of a cohort of PWH in Rio de Janeiro, Brazil, through the analysis of medications prescribed during the year 2019. Almost 80% of PWH were using efavirenz or dolutegravir, ARV recommended as first-line regimen by the Brazilian National Guidelines, and PWH ≥ 50 years were more frequently using PI or etravirine, ARV recommended as second- or third-line regimens [21]. Almost half of PWH were experiencing polypharmacy, and most frequently concomitant medications groups prescribed were nervous system, antiinfectives for systemic use, alimentary tract and metabolism and cardiovascular. Regression model results indicated that the odds of polypharmacy increased with age, as well as among cisgender women, those with lower education and those who were living with HIV for more than 10 years.

Our first result of importance to HIV care refers to the significant aging of the individuals in care at our institution. In 2008, median age of PWH (n = 2307) in care was 33.1 (IQR: 27.3–40.2) [22], while in the present study it has increased to 44.4 (IQR: 35.4–54.1). Likewise, we note a decreased proportion of younger and increased proportion of older PWH between 2008 and 2019 (18–39 years: 44.3% vs. 38.0%; 40–49 years: 35.7% vs. 26.5%; 50–59 years: 15.3% vs. 23.3%, and ≥ 60 years: 4.7% vs. 12.2% respectively). Similar results have been shown globally using data from the International Epidemiology Databases to Evaluate AIDS (IeDEA) consortium [23] and may be explained by the increased in life expectancy provided by ARV [6].

Older PWH (≥ 50 years) received more prescriptions of PI drugs than younger participants (< 50 years). This may be result from the higher probability of older PWH being on second- and third-line regimens, as most are living with HIV for longer time than younger PWH. Medium and long-term use of PI may lead to metabolic syndrome which is associated with increased risk of cardiovascular diseases. These PI-related issues need to be closely monitored as PI drugs are metabolized by cytochrome P450, are more susceptible to drug-drug interaction, and may interact with hypertension medications, such as calcium channel blockers and beta blockers [24]. Older PWH being more susceptible to PI use and to experience polypharmacy is a sensitive issue in the evaluation of prescriptions to PWH.

In our study, prescribed nervous system medications were common among all age strata, similar to observed in a large study conducted in Spain [18]. The high number of prescriptions of nervous system medications may be related to neurologic complications related to HIV/AIDS, such as cognitive impairment [25], which is estimated to affect almost half of all PWH in the world (~ 18 million). In addition, 31.4% of individuals in this study were using efavirenz, an ARV frequently linked to neuropsychiatric adverse events [26]. Although ARV initiation may be enough to prevent new nervous system damages, existing abnormalities caused by the virus are irreversible [27]. HIV infection can trigger the entry of monocyte-derived macrophages into the central nervous system (CNS) across the blood-brain barrier, releasing cytokines and viral products that can break endothelial barriers and affect neural pathways. HIV can also promote virus replication in compartmental areas of the CNS leading to dysregulation of neural pathways [28]. In addition to physiological issues, stigma against PWH can lead to the development of psychological disorders in PWH, such as depression, anxiety, sudden mood swings, which may increase nervous system medications use [29].

Cardiovascular system medications were frequently prescribed among older individuals (≥ 50 years), similar to observed among older PWH in high income settings, such as United Kingdom [30] and Switzerland [31, 32]. Still, there is no consensus whether HIV and ARV are directly associated with cardiovascular diseases. Data indicate that prevalence of systemic arterial hypertension is higher among PWH, especially among the oldest [33]. In a study conducted in Sub-Saharan Africa, longer time in ARV and increased age were associated to increased carotid intima-media thickness which indicated higher risk of cardiovascular disease [34]. In addition to infectious disease specialist care, a cardiology specialist must periodically review the medications of PWH, to correctly plan the combination of cardiovascular system medications and ARV, avoiding potential interactions. In the Public Health System, the infectious disease specialist may also perform initial cardiovascular risk screening before referring the patient to a cardiology specialist.

The prevalence of alimentary tract and metabolism medications was high in our study. Microvilli atrophy processes, abscesses, malabsorption, poor digestion, abdominal pain and nausea are common among PWH affecting 50–70% globally [35]. These conditions can be caused by a direct action of HIV on intestinal cells and local immunological changes, facilitating opportunistic pathogens (e.g. fungi, bacteria, parasites and protozoa) infection that can lead to diarrhea and chronic malnutrition, significantly affecting individuals quality of life [36]. Although ARV has greatly reduced the incidence of opportunistic diseases in PWH, including in our cohort [37], *Helicobacter pylori* infections are still common in this population and are associated with gastroesophageal reflux disease. Data suggests that an immune improvement through the increase in CD4 cells provided by ARV allows for a more effective inflammatory response that could provide favorable conditions for *Helicobacter pylori* growth by increasing stomach acid secretion (40).

Half of individuals included in in this study were experiencing polypharmacy, twice higher than found in a study conducted in Minas Gerais, Brazil that included less individuals aged ≥ 50 years (23% vs. 36% in the present study) [16]. Studies conducted in low and high income settings among PWH found slightly lower prevalence of polypharmacy (~ 40%) [14, 15, 30]. Conversely, prevalence of polypharmacy in an Australian study conducted in 2018 with 552 participants using ARV was slightly higher than observed in this study (54%) [38], which may be related to the higher median time since HIV diagnosis compared to our study (15 years vs. 10 years).

Polypharmacy increased with age and time living with HIV, as observed in other studies [18, 30, 32]. This is in accordance with the profile of morbidities commonly found among individuals aged ≥ 50 years, which can probably be linked to the deterioration of physiological conditions resulting from advancing age and long term effects of HIV infection [8]. Even PWH on ARV with undetectable viral load may be impacted by the chronic inflammation caused by HIV. This process is characterized by the presence of short telomeres, indicating an acceleration of cell aging and an increased risk of developing comorbidities [39]. ARV long-term toxicity can disrupt several cellular processes, including autophagy, leading to cellular dysfunction linked to premature aging [11]. The possibility of cascading prescription that arises from the attempt to treat new clinical conditions triggered by adverse effects of chronic medication use, especially ARV, lead to the prescription of additional medications [12]. To this end, knowing the pharmacotherapeutic profile of our population is essential for deprescription, especially among older individuals. In a randomized clinical trial conducted in the United States, pharmacist-led review of medication prescriptions revealed a large number of potentially inappropriate prescribing, many amenable to immediate clinical pharmacist intervention [40].

Compared to cisgender man, cisgender woman had increased odds of polypharmacy. According to a study conducted in Bahia, Brazil, higher prevalence of polypharmacy among cisgender woman may be related to longer survival, higher demand for health services and higher familiarity with medication [41]. In a large study conducted in Spain, polypharmacy was also more frequent among cisgender woman living with HIV [18]. According to the authors, a more frequent contact with the health system by cisgender women can provide them an extra opportunity to diagnose diseases and receive prescriptions [18]. Other hypotheses include biological factors related to gender and differences in the occurrence of specific comorbidities associated with chronic medication use [42, 43] and that women are also more likely to participate in preventive health care, thus having a greater possibility of receiving prescriptions for drugs that are used for disease prevention [44]. Our results also identified an association of low education and polypharmacy that can be related to socioeconomic disparities, influencing access to healthy food and basic health services, which are elements that have a great impact on quality of life and can lead to the development of diseases [12, 45].

This study has limitations. Only prescriptions generated through the INI-Fiocruz system were included in our study. Therefore, medications prescribed by physicians outside of INI-Fiocruz or self-medication were not considered. Additionally, prescriptions do not assure drug refill or use. We considered all medications prescribed during the year 2019 without further assessment of when, within the year, these medications were used. As such, we cannot guarantee simultaneous use of all medications. One strength of our study is the large source population of INI-Fiocruz, that includes PWH since 1986, providing a unique opportunity to identify and monitor the evolution of the most common comorbidities that affect PWH in care, as well as the medication classes most used in this population. Our findings are useful for planning and creating more efficient strategies that allow a better management of the therapy, adherence, and effectiveness, as well as avoiding possible adverse drug-related event.

In conclusion, high rates of polypharmacy were found in a cohort of PWH in Brazil. Access to ARV increased the survival of PWH, which is more exposed to age-related chronic diseases and, consequently, increased use of medications to treat these comorbidities. Targeted interventions to prevent interactions and improve treatment adherence are warranted, especially among certain groups, such as cisgender women, older individuals, and those with lower education. Standardized protocols for continuous review of patients’ therapeutic regimens should be implemented.

### Electronic supplementary material

Below is the link to the electronic supplementary material.

Supplementary Material 1

## Data Availability

The datasets used and/or analyzed during the current study are available from the corresponding author on reasonable request.

## List of Abbreviations

aOR: Adjusted odds ratio
ARV: Antiretroviral drugs
ATC: Anatomical Therapeutic Chemical
IF: Fusion inhibitor
IQR: Interquartile range
INI/Fiocruz: Instituto Nacional de Infectologia Evandro Chagas, Fundação Oswaldo Cruz
INSTI: Integrase strand transfer inhibitors
NNRTI: Non-nucleoside reverse transcriptase inhibitors
NRTI: Nucleoside reverse transcriptase inhibitors
OR: Odds ratio
PEP: Post-exposure prophylaxis
PI: Protease inhibitors
PrEP: Pre-exposure prophylaxis
PWH: People living with HIV
SUS: Sistema Único de Saúde [Brazilian Unified Health System
UNAIDS: Joint United Nations Programme on HIV/AIDS
WHO: World Health Organization
