# Supplementary material for: Pharmacotherapeutic profile, polypharmacy and its associated factors in a cohort of people living with HIV in Brazil
Source: AIDS Res Ther. 2023 Aug 21;20:57. doi: 10.1186/s12981-023-00548-6 (PMC10440883; doi:10.1186/s12981-023-00548-6)
Supplement: Supplementary file 1 — Supplementary Material 1 [file 12981_2023_548_MOESM1_ESM.docx]

**Supplementary Table 1**. **Proportion of participants who received prescriptions of concomitant medications according to ATC subgroup code stratified by age (<50 vs. ≥ 50 years) in a referral center in Rio de Janeiro, Brazil, 2019 (N=4547).**

|  | **Total** | **< 50 years** | **≥ 50 years** | **P value** |
| --- | --- | --- | --- | --- |
|  | N= 4547 | N= 2932 | N= 1615 |  |
| **A. Alimentary Tract & Metabolism** | 2087 (45.9) | 1179 (40.2) | 908 (56.2) | < 0.001 |
| A02. Acid related disorders | 1057 (23.2) | 563 (19.2) | 494 (30.6) | < 0.001 |
| A03. Functional gastrointestinal disorders | 1162 (25.6) | 700 (23.9) | 462 (28.6) | < 0.001 |
| A04. Antiemetics and antinauseants | 292 (6.4) | 194 (6.6) | 98 (6.1) | 0.47 |
| A06. Constipation | 312 (6.9) | 155 (5.3) | 157 (9.7) | < 0.001 |
| A07. Antidiarrheals, intestinal anti-inflammatory / anti-infective | 274 (6.0) | 156 (5.3) | 118 (7.3) | 0.007 |
| A10. Diabetes | 408 (9.0) | 136 (4.6) | 272 (16.8) | < 0.001 |
| A11. Vitamins | 155 (3.4) | 110 (3.8) | 45 (2.8) | 0.086 |
| A12. Mineral supplements | 73 (1.6) | 44 (1.5) | 29 (1.8) | 0.449 |
|  |  |  |  |  |
| **B. Blood & Blood Forming Organs** | 1085 (23.9) | 545 (18.6) | 540 (33.4) | < 0.001 |
| B01. Antithrombotic | 500 (11.0) | 162 (5.5) | 338 (20.9) | < 0.001 |
| B02. Antihemorrhagic | 15 (0.3) | 10 (0.3) | 5 (0.3) | 0.859 |
| B03. Antianemia | 426 (9.4) | 252 (8.6) | 174 (10.8) | 0.016 |
| B05. Blood substitutes and perfusion solutions | 496 (10.9) | 317 (10.8) | 179 (11.1) | 0.778 |
|  |  |  |  |  |
| **C.** C**ardiovascular System** | 1821 (40.0) | 740 (25.2) | 1081 (66.9) | < 0.001 |
| C01. Cardiac therapy | 102 (2.2) | 34 (1.2) | 68 (4.2) | < 0.001 |
| C02. Antihypertensives | 139 (3.1) | 42 (1.4) | 97 (6.0) | < 0.001 |
| C03. Diuretics | 507 (11.2) | 182 (6.2) | 325 (20.1) | < 0.001 |
| C04. Peripheral vasodilators | 19 (0.4) | 3 (0.1) | 16 (1) | < 0.001 |
| C07. Beta blocking agents | 357 (7.9) | 99 (3.4) | 258 (16) | < 0.001 |
| C08. Calcium channel blockers | 234 (5.1) | 63 (2.1) | 171 (10.6) | < 0.001 |
| C09. Agents acting on the renin-angiotensin system | 918 (20.2) | 299 (10.2) | 619 (38.3) | < 0.001 |
| C10. Lipid modifying agents | 1245 (27.4) | 452 (15.4) | 793 (49.1) | < 0.001 |
|  |  |  |  |  |
| **D. Dermatologicals** | 1431 (31.5) | 843 (28.8) | 588 (36.4) | < 0.001 |
| D01. Antifungals for dermatological use | 551 (12.1) | 344 (11.7) | 207 (12.8) | 0.283 |
| D02. Emollients and protectives | 180 (4) | 105 (3.6) | 75 (4.6) | 0.079 |
| D03. Treatment of wounds and ulcers | 73 (1.6) | 38 (1.3) | 35 (2.2) | 0.025 |
| D06. Antibiotics and chemotherapeutics for dermatological use | 474 (10.4) | 293 (10) | 181 (11.2) | 0.2 |
| D07. Corticosteroids, dermatological preparations | 700 (15.4) | 391 (13.3) | 309 (19.1) | < 0.001 |
| D08. Antiseptics and disinfectants | 17 (0.4) | 14 (0.5) | 3 (0.2) | 0.123 |
| D09. Medicated dressing | 109 (2.4) | 74 (2.5) | 35 (2.2) | 0.452 |
| D11. Other dermatological preparations | 2 (0) | 2 (0.1) | 0 (0) | 0.294 |
|  |  |  |  |  |
| **G. Genito Urinary System & Sex Hormones** | 413 (9.1) | 311 (10.6) | 102 (6.3) | < 0.001 |
| G01. Gynecological antiinfectives and antiseptics | 280 (6.2) | 196 (6.7) | 84 (5.2) | 0.046 |
| G03. Sex hormones and modulators of the genital system | 168 (3.7) | 156 (5.3) | 12 (0.7) | < 0.001 |
| G04. Urological | 13 (0.3) | 4 (0.1) | 9 (0.6) | 0.011 |
|  |  |  |  |  |
| **H. Systemic Hormonal Preparations, excluding Sex Hormones and Insulins** | 642 (14.1) | 368 (12.6) | 274 (17.0) | < 0.001 |
| H01. Pituitary and hypothalamic hormones and analogues | 25 (0.5) | 10 (0.3) | 15 (0.9) | 0.01 |
| H02. Corticosteroids for systemic use | 567 (12.5) | 351 (12) | 216 (13.4) | 0.17 |
| H03. Thyroid therapy | 86 (1.9) | 23 (0.8) | 63 (3.9) | < 0.001 |
|  |  |  |  |  |
| **J. Antiinfectives for Systemic Use** | 2728 (60.0) | 1830 (62.4) | 898 (55.6) | < 0.001 |
| J01. Antibacterials | 1838 (40.4) | 1266 (43.2) | 572 (35.4) | < 0.001 |
| J02. Antimicotics | 441 (9.7) | 314 (10.7) | 127 (7.9) | 0.002 |
| J04. Antimicobacterials | 255 (5.6) | 203 (6.9) | 52 (3.2) | < 0.001 |
| J05. Antivirals | 421 (9.3) | 259 (8.8) | 162 (10.0) | 0.182 |
| J06. Immune sera and immunoglobulins | 7 (0.2) | 7 (0.2) | 0 (0) | 0.049 |
| J07. Vaccines | 1028 (22.6) | 702 (23.9) | 326 (20.2) | 0.004 |
|  |  |  |  |  |
| **L. Antineoplasic & Immunomodulating Agents** | 53 (1.2) | 44 (1.5) | 9 (0.6) | 0.005 |
| L01. Antineoplastic | 30 (0.7) | 27 (0.9) | 3 (0.2) | 0.003 |
| L03. Imunostimulants | 26 (0.6) | 21 (0.7) | 5 (0.3) | 0.082 |
| L04. Imunossupressants | 9 (0.2) | 7 (0.2) | 2 (0.1) | 0.404 |
|  |  |  |  |  |
| **M. Musculo-skeletal System** | 1126 (24.8) | 665 (22.7) | 461 (28.5) | < 0.001 |
| M01. Antiinflammatory and antirheumatic | 1005 (22.1) | 626 (21.4) | 379 (23.5) | 0.1 |
| M02. Topical products for joint and muscular pain | 8 (0.2) | 3 (0.1) | 5 (0.3) | 0.11 |
| M03. Muscle relaxants | 87 (1.9) | 42 (1.4) | 45 (2.8) | 0.001 |
| M04. Antigout preparations | 88 (1.9) | 28 (1) | 60 (3.7) | < 0.001 |
| M05. Drugs for treatment of bone diseases | 13 (0.3) | 2 (0.1) | 11 (0.7) | < 0.001 |
|  |  |  |  |  |
| **N. Nervous System** | 2947 (64.8) | 1766 (60.2) | 1181 (73.1) | < 0.001 |
| N01. Anesthetics | 125 (2.7) | 90 (3.1) | 35 (2.2) | 0.075 |
| N02. Analgesics | 2521 (55.4) | 1509 (51.5) | 1012 (62.7) | < 0.001 |
| N03. Antiepileptics | 959 (21.1) | 555 (18.9) | 404 (25.0) | < 0.001 |
| N04. Anti-parkinson | 18 (0.4) | 7 (0.2) | 11 (0.7) | 0.023 |
| N05. Psycholeptics | 532 (11.7) | 290 (9.9) | 242 (15) | < 0.001 |
| N06. Psychoanaleptics | 875 (19.2) | 461 (15.7) | 414 (25.6) | < 0.001 |
| N07. Other nervous system drugs | 68 (1.5) | 28 (1) | 40 (2.5) | < 0.001 |
|  |  |  |  |  |
| **P. Antiparasitic, Insecticides & Repellents** | 621 (13.7) | 432 (14.7) | 189 (11.7) | 0.004 |
| P01. Antiprotozoal | 211 (4.6) | 147 (5.0) | 64 (4.0) | 0.107 |
| P02. Anthelmintics | 490 (10.8) | 341 (11.6) | 149 (9.2) | 0.012 |
| P03. Ectoparasiticides, including scabicides, insecticides and repellents | 56 (1.2) | 38 (1.3) | 18 (1.1) | 0.595 |
|  |  |  |  |  |
| **R. Respiratory System** | 1685 (37.1) | 998 (34.0) | 687 (42.5) | < 0.001 |
| R02. Throat preparations | 43 (0.9) | 27 (0.9) | 16 (1.0) | 0.816 |
| R03. Drugs for obstructive airway diseases | 739 (16.3) | 421 (14.4) | 318 (19.7) | < 0.001 |
| R05. Cough and cold preparations | 891 (19.6) | 492 (16.8) | 399 (24.7) | < 0.001 |
| R06. Antihistamines for systemic use | 950 (20.9) | 587 (20) | 363 (22.5) | 0.051 |
|  |  |  |  |  |
| **S. Sensory Organs** | 262 (5.8) | 140 (4.8) | 122 (7.6) | < 0.001 |
| S01. Ophthalmologicals | 251 (5.5) | 133 (4.5) | 118 (7.3) | < 0.001 |
| S03. Ophthalmological and ontological preparations | 21 (0.5) | 11 (0.4) | 10 (0.6) | 0.245 |
|  |  |  |  |  |
| **V. Various** | 135 (3.0) | 71 (2.4) | 64 (4.0) | 0.003 |
| V03. All other therapeutic products | 125 (2.7) | 64 (2.2) | 61 (3.8) | 0.002 |
| V09. Diagnostic radiopharmaceuticals | 11 (0.2) | 8 (0.3) | 3 (0.2) | 0.567 |

ATC: Anatomical Therapeutic Chemical classification system.
